# Familial risk of early- and late-onset multiple sclerosis: a Swedish nationwide study

**DOI:** 10.1007/s00415-018-9163-6

**Published:** 2018-12-21

**Authors:** Jie Song, Helga Westerlind, Kyla A. McKay, Catarina Almqvist, Pernilla Stridh, Ingrid Kockum, Jan Hillert, Ali Manouchehrinia

**Affiliations:** 1 Department of Medical Epidemiology and Biostatistics, Karolinska Institutet, Stockholm, Sweden; 2 Unit of Cardiovascular Epidemiology, Institute of Environmental Medicine, Karolinska Institutet, Stockholm, Sweden; 3 Clinical Epidemiology Division, Department of Medicine, Solna, Karolinska Institutet, Stockholm, Sweden; 4 Centre for Molecular Medicine, Department of Clinical Neuroscience, Karolinska Institutet, Stockholm, Sweden; 5 Lung and Allergy Unit, Astrid Lindgren Children’s Hospital, Karolinska University Hospital, Stockholm, Sweden; 6 Department of Clinical Neuroscience, The Karolinska Neuroimmunology and Multiple Sclerosis Centre, Centre for Molecular Medicine, Karolinska Institutet, Stockholm, Sweden

**Keywords:** Multiple sclerosis, Familial risk, Genetics, Early onset, Late onset

## Abstract

**Background:**

Persons who develop multiple sclerosis (MS) at a young age may bear a higher genetic risk load than persons who develop MS later in life; however, the contribution of familial influence to the risk of MS, in relation to onset age, has not been established.

**Objective:**

To investigate the familial risk of MS at two extremes of the spectrum of MS onset age: early onset (first MS symptom < 18 years of age) and late onset (first MS symptom ≥ 50 years).

**Methods:**

Nationwide registries in Sweden were used to identify cases of MS and controls, and their familial relations. We estimated the odds ratio (OR) of an MS diagnosis for individuals with a relative diagnosed with early-onset or late-onset MS compared with those whose relatives did not have MS, using a nested case–control design.

**Results:**

629 early-onset and 1148 late-onset MS patients were identified and matched to 10 controls from the general population by age and sex. The OR of MS for individuals with a first-degree relative diagnosed with early-onset MS was 10.86 (95% CI 6.87–17.17); and for late-onset MS was 8.08 (95% CI 6.12–10.67).

**Conclusions:**

Our findings demonstrate no substantial differences in familial risk in persons with early- and late-onset MS.

## Introduction

The underlying aetiology of multiple sclerosis (MS) is unknown, but likely involves an interaction between genes and environment [1]. The genetic risk of the disease appears to be largely driven by alleles controlling the human leukocyte antigen (HLA) system. Of the many environmental factors assessed, cigarette smoking, Epstein–Barr virus seropositivity, infectious mononucleosis, and low vitamin D/sunlight exposure have shown the most consistent associations [2–4]. Though not considered a hereditary disease, MS can aggregate in families and the estimated recurrence risks for MS in Sweden are 17.3% for monozygotic twins and 2.6% for siblings [5].

The first neurological symptom suggestive of MS typically occurs between the ages of 20 and 40 [6]. Early (≤ 18 years) and late (≥ 50 years) onset of MS are less common, with prevalence estimates between 2–10% and 4–9%, respectively [7, 8]. Previous studies have reported that carriers of the allele HLA-DRB1*15 develop the disease earlier than non-carriers [9–11], indicating a potential genetic role in the timing of disease onset. Studies of environmental factors have suggested shared risk factors between childhood- and adult-onset disease, including exposure to tobacco smoke, Epstein–Barr virus infection, and obesity [12]. Further, there are shared genetic risk factors between childhood- and adult-onset disease, with HLA-DRB1*15 and 28 out of 104 tested non-MHC variants (23%) associated to MS in the early-onset group [13]. Studies of familial risks offer a unique opportunity to investigate the aetiology of MS, as the familial risk constitutes both genetic and environmental risk factors shared within a family. The relationship between familial risk and age at MS onset could have a profound effect on our understanding of the aetiology of MS. In this study, we investigated the familial risk of MS at the two extremes of the spectrum of MS onset age: early-onset MS (EOMS) and late-onset MS (LOMS).

## Methods

### Study population and ascertainment of MS cases

The population-based cohort was established through the linkage of a series of nationwide Swedish registers using the unique identification numbers assigned to all residents of Sweden. The National Patient Register (NPR) contains diagnoses according to the International Classification of Disease (ICD) on all inpatient (from 1968) and outpatient (from 2001) visits in Sweden [14]. The Multi-generation Registry contains information on parents and adoptive parents for all persons born in Sweden since 1932 and registered as living in Sweden since January 1, 1961. The registry allows for the identification of familial relations, including first-degree (parents, offspring, and siblings) and second-degree relatives (grandparents, grandchildren, uncles/aunts, nephews/nieces, and half-siblings). The Swedish MS Register (SMSreg) is a nationwide MS-specific quality register used by all 64 neurology clinics in Sweden. It began in 2001 and contains demographic and clinical data on an estimated 85% of all prevalent cases of MS in Sweden [15]. The Total Population, Migration and Cause of Death Registry provided demographic data and death records for Swedish residents and enabled us to ensure that persons were alive and reside in the country at the time of matching. Data from all registries were available until December 31, 2013. This study was approved by the Stockholm regional ethical committee at Karolinska Institutet and has been performed in accordance with the ethical standards laid down in the 1964 Declaration of Helsinki and its later amendments.

EOMS and LOMS were defined as MS onset < 18 or ≥ 50 years of age, respectively. As information on onset date was only available from the SMSreg, these cases were all derived from the SMSreg. Date of MS onset is recorded by a MS specialist neurologist following confirmation of clinically definite MS diagnosis. The MS status of relatives of the probands was established using the SMSreg and NPR, as not all persons with MS in Sweden are captured in the SMSreg, specifically for the old cases. Therefore, persons with ≥ 2 diagnostic codes specific for MS [ICD-8 (340), ICD-9 (340) or ICD-10 (G35)] or who were registered in the SMSreg as having definite MS, were classified as having MS. Two or more ICD codes were chosen as our previous investigation suggested that false positive MS diagnosis belong almost exclusively to the patients having only one confirmation of MS [16].

### Statistical analyses

We employed a nested case–control design to examine the risk of being diagnosed with MS in relatives of patients with EOMS/LOMS, compared with being diagnosed with MS in relatives of general population controls. This design is also called “incidence density sampling”, because it enables the estimation of incidence rates. Compared to case–control studies which estimate the ratio of odds of prevalence, nested case–control study estimates the ratio of odds of incidence. The control group consisted of up to ten individuals randomly selected from the Total Population Register and matched to each case on sex and birth year. The ten individuals were required to be alive, reside in Sweden and have no MS diagnosis by the date of the index case’s onset of MS (a 2-year delay was allowed for a possible lag in diagnosis). Their relatives were also matched to the case’s relative by sex, year of birth, and biological relationship. More details about this method can be found in previous publications [5, 17].

We fit a conditional logistic regression model to examine the odds ratios (ORs) in offspring, siblings, grandparents, grandchildren, uncles/aunts, nephews/nieces, and half-siblings of the probands. To increase power, we merged all first-degree relatives and second-degree relatives (separately) and estimated the odds ratios for each group. Only probands were selected by age of onset, the relatives were selected to be affected by MS or not affected regardless of age at onset. This is due to consideration that EOMS and LOMS are so rare that a relative pair of both EOMS/LOMS would be even rarer and also that we are interested in estimating the increased/decreased risk of MS in general not only EOMS or LOMS. The 95% confidence intervals (CIs) were calculated with standard errors supplied by a robust sandwich estimator that corrects for non-independence due to familial clustering. Bonferroni correction was applied to account for multiple testing (*N* = 11), and a *P* value < 0.005 was considered statistically significant. A significantly increased OR would suggest that familial risk factors shared among relatives contribute to the association between MS and EOMS/LOMS. Comparable ORs between the two groups would suggest a similar magnitude of contributions from familial risk factors. All analyses were performed using SAS version 9.4.

## Results

**Fig. 1:**
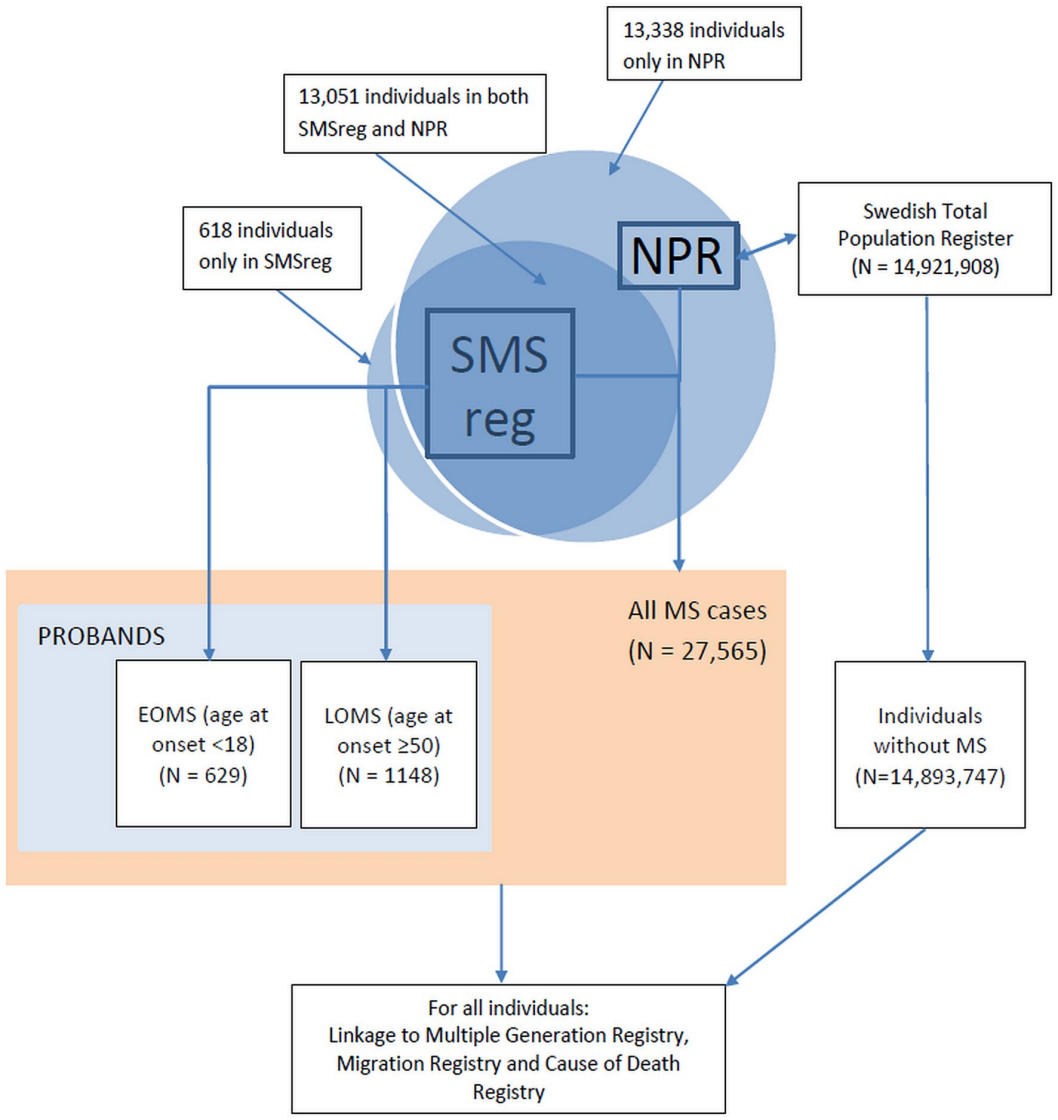
Flowchart of data collection in Swedish national registries. *MS* multiple sclerosis, *EOMS* early-onset multiple sclerosis, *LOMS* late-onset multiple sclerosis, *NPR* National Patient Register, *SMSreg* Swedish MS registry

Demographic information for the EOMS and LOMS groups is presented in Table 1. Figure 1 illustrates the study diagram and number of persons extracted from each of the registries.

**Table 1:** Demographic characteristics of the MS study population

	Age at onset < 18 year (EOMS)	Age at onset ≥ 50 year (LOMS)
Number of patients	629	1148
Mean age at onset, years (SD)	15.5 (2.2)	55.5 (4.7)
Mean calendar year of birth (SD)	1974 (16.9)	1946 (8.1)
Mean calendar year at onset (SD)	1990 (16.8)	2002 (7.2)
Number of females (%)	453 (72.0)	774 (67.4)

*EOMS* early -onset multiple sclerosis, *LOMS* late-onset multiple sclerosis, *SD* standard deviation

We identified 629 patients with an MS onset before age 18 (EOMS) and 1148 whose onset age was greater than or equal to 50 years (LOMS) from the SMSreg. The mean calendar year of birth was 1946 among the LOMS cohort and 1974 among the EOMS.

There were 26,884 persons who met the NPR definition of MS (≥ 2 ICD codes for MS); 13,051 of whom were also registered in the SMSreg as having definite MS. An additional 681 persons were identified from the SMSreg who did not meet the NPR definition, for a total of 27,565 unique MS cases identified (Fig. 1).

A significant risk for developing MS was observed among first- and second-degree relatives of individuals in both groups (Table 2). The ORs of MS for individuals who had a first- or second-degree relative with EOMS were 10.86 (95% CI 6.87–17.17) and 3.83 (95% CI 2.48–5.90), respectively. The corresponding ORs for individuals with a first- or second-degree relative diagnosed with LOMS were 8.08 (95% CI 6.12–10.67) and 2.86 (95% CI 1.87–4.39), respectively. Adjusting for sex and stratum of birth year (< 1948, 1948–1963, 1964–1985, ≥ 1986) did not alter the results (data not shown). The overlapping confidence intervals suggested no significant differences in the ORs of MS for relatives of EOMS and LOMS patients.

**Table 2:** Odds ratios of multiple sclerosis in relatives of patients with early- or late-onset multiple sclerosis

Relationship to proband	MS in individuals with a relative with EOMS				MS in individuals with a relative with LOMS			
	Proband group with EOMS (%)	Matched control group (%)	*P* value	OR (95% CI)	Proband group with LOMS (%)	Matched control group (%)	*P* value	OR (95% CI)
**First-degree relative**	44 (1.8)	41 (0.2)	< 0.001	10.86 (6.87–17.17)	104 (1.8)	130 (0.2)	< 0.001	8.08 (6.12–10.67)
Parent	29 (2.6)	24 (0.2)	< 0.001	12.43 (7.79–19.83)	22 (1.1)	32 (0.2)	< 0.001	6.99 (4.38–11.16)
Offspring	5 (0.9)	5 (0.1)	< 0.001	11.50 (3.75–35.26)	33 (1.5)	43 (0.2)	< 0.001	7.78 (5.22–11.58)
Full sibling	10 (1.3)	12 (0.2)	< 0.001	8.33 (3.88–17.88)	49 (3.1)	55 (0.4)	< 0.001	9.11 (6.33–13.10)
**Second-degree relative**	32 (0.8)	84 (0.2)	< 0.001	3.83 (2.48–5.90)	26 (0.4)	91 (0.1)	< 0.001	2.86 (1.87–4.39)
Grandparent	11 (0.8)	30 (0.2)	< 0.001	3.67 (2.01–6.70)	1 (0.5)	1 (0.1)	0.06	10.00 (0.94–105.9)
Grandchild	0 (0.0)	0 (0.0)	–	–	2 (0.1)	11 (0.0)	0.40	1.82 (0.45–7.33)
Uncle/aunt	17 (1.3)	41 (0.3)	< 0.001	4.15 (2.44–7.06)	2 (1.9)	3 (0.3)	0.02	6.67 (1.44–30.94)
Nephew/niece	0 (0.0)	8 (0.1)	–	–	21 (0.7)	68 (0.2)	< 0.001	3.10 (1.96–4.91)
Paternal half-sibling	1 (0.6)	4 (0.2)	0.37	2.50 (0.34–18.27)	0 (0.0)	4 (0.3)	–	–
Maternal half-sibling	3 (2.1)	1 (0.1)	0.001	30.0 (3.84–233.8)	0 (0.0)	4 (0.5)	–	–

Case:control = 1:10 matched on age and sex. Number of MS cases in relatives of a person diagnosed with EOMS, LOMS and a healthy control are shown with percentages in brackets

*MS* multiple sclerosis, *OR* odds ratio, *EOMS* early-onset multiple sclerosis, *LOMS* late-onset multiple sclerosis

The *P* value and OR represent results from a comparison between the proband and matched control groups

## Discussion

In this nationwide study of early- and late-onset MS cases in Sweden, we found similar familial risks between those who developed MS early (age < 18 years) and those who developed the disease late in life (50+ years), indicating a persistent familial risk, regardless of the timing of MS clinical manifestation.

Instinctively, a higher familial risk is thought to compensate for the shorter time of exposure to the environmental trigger(s) in EOMS patients. While we found slightly higher odds of having a family member with MS in the EOMS cohort, the odds of the LOMS cohort were not significantly different, and so this notion could not be supported by our findings.

Two previous studies have explored sibling risk in relation to age at MS onset and both found a strong influence of onset age on recurrent risk of MS [18, 19]. Recent work has explored the effect of familial risk on disease subtype and across unique cohorts [5]. In a similar approach, we previously compared the cerebrospinal fluid oligoclonal band status (positive or negative) in MS and found no familial risk differences between groups [17]. Both of our previous studies on familial risks [5, 17] and a population-based study in Denmark [20] reported no difference in familial risk between male and female probands. Taken together, these results suggest a relatively homogenous effect of familial risk across disease subtypes and cohorts of patients. Genetic studies have been performed more extensively. A recent study of genetic risk of childhood-onset MS reported a higher frequency of DRB1*15:01 in paediatric onset cases compared with adult-onset disease, while similar genetic association to variants outside the HLA were detected for early- and adult-onset MS [21]. Previous studies have also reported association between HLA-DRB1*15 and younger onset age [10, 22]. When investigating the association between DRB1*15:01 and SNPs in LD, and age at onset, each DRB1*15:01 allele was associated with 10.6 months reduction in age at onset with no other SNPs showing strong evidence of association [23]. Similarly, studies of environmental factors suggest shared risk factors between childhood- and adult-onset disease [12]. Many of the risk factors for adult-onset MS appear to have critical periods during adolescence [24, 25]. Given the overlap in the familial risks between the early- and late-onset patients found here, it remains unknown why some people develop the disease early in life and others late in life. Quite possibly, there are a number of different sufficient causes of MS, with similar genetic contributions that initiate the disease process in each onset age group [26]. The environmental contributions can be either non-shared or a shared overlapping collection. It is unknown why disease onset is relatively rare in children, especially given these similar risk factors.

Strengths of this study include the large, population-based cohort and longitudinal design. While our study included over 600 EOMS patients, which is substantial in the context of childhood-onset MS observational studies, it is possible that the sample was insufficiently powered to detect a difference between groups. Second, it is known that the biological onset of MS likely occurs many years prior to the clinical onset of disease, and the definition of onset requires patient recall, which may become less accurate with time. It is possible that some late-onset cases had an earlier onset, but were not identified until later life. Third, given the difference in calendar year of birth between the two groups, we had a structural limitation in identifying second-degree relatives (specifically grandchildren in the early-onset group and grandparents in the late-onset group). This makes the estimates in specific strata of second-degree relatives less comparable between EOMS and LOMS. Nevertheless, the overall familial risks in the second-degree relatives of the two groups did not show a significant difference and were comparable with the risk in general MS [17]. It is likely that proportion of cases with primary progressive MS phenotype is higher in the LOMS group which potentially could have different genetic backgrounds. Unfortunately, data on MS phenotype were not available in this linkage. Further, we identified MS in familial relations using hospital and physician billings for MS. As this is not a formal diagnosis, it is possible that some persons were misclassified. Nevertheless, the health administrative data offers a valuable resource to conduct sizable research studies in samples representative of populations. In addition, we expect that any diagnosis misclassification would not bias our results between groups, but may lead to some imprecision in the risk estimates. Last, the Swedish population has a unique genetic composition and the environmental exposures which contribute to MS risk within Sweden may not be generalizable to populations outside of this region.

In conclusion, our study supports the strong familial influence on MS risk; however, we found no evidence of a familial effect on the age at which MS first manifests.
